# Fabrication of novel electropolymerized conductive polymer of hydrophobic perfluorinated aniline as transducer layer on glassy carbon electrode: application to midazolam as a model drug of benzodiazepines

**DOI:** 10.1186/s13065-023-00945-y

**Published:** 2023-04-04

**Authors:** Ekram H. Mohamed, Amr M. Mahmoud, Nancy W. Nashat, Sally S. El-Mosallamy

**Affiliations:** 1 Pharmaceutical Analytical Chemistry Department, Faculty of Pharmacy, The British University in Egypt, El-Shorouk City, 11837 Egypt; 2 Analytical Chemistry Department, Faculty of Pharmacy, Cairo University, Kasr El Aini, Cairo, 11562 Egypt

**Keywords:** Solid-contact ion-selective electrodes, Hydrophobic polymers, Perfluorinated polyaniline, Midazolam, Glassy carbon electrodes

## Abstract

The objective of this study is to fabricate solid-contact ion selective electrodes (SC-ISEs) that have long term stable potential. Various conducting polymers such as polyaniline and its derivatives have been successfully employed to improve the potential stability in SC-ISEs. Recently, the role of hydrophobicity at the interface between the conducting polymer solid contact and the ion sensing membrane has been investigated and figured out that the hydrophobic interfaces preclude water layer formation that deteriorate the SC-ISEs potential stability and reproducibility. In this work, a hydrophobic polyaniline derivative was fabricated on the surface of a glassy carbon electrode by electropolymerization of perfluorinated aniline monomers in acidic solution. The electropolymerized hydrophobic polymer was characterized by electrochemical impedance spectroscopy and X-ray photoelectron spectroscopy. The fabricated electrode was employed for determination of midazolam—a model drug-in pharmaceutical formulation without prior extraction. The SC-ISEs performance was optimized, and the potential drift was compared to control SC-ISEs, the SC-ISE linear range was 1 × 10^–6^–1 × 10^–2^ M, LOD was estimated to be 9.0 × 10^–7^ M, and potential drift was reduced to 100 μV/h.

## Introduction

Electrochemical determination of drugs have thrived in the recent years leaving an impact in various fields such as medicinal, environmental [1, 2], industrial, agricultural [3] and pharmaceutical [4–6]. Ion-selective electrodes (ISEs), especially the solid contact ones, have been successfully utilized in point of care devices. This is attributed to the need for simple, portable and reliable devices. This explains why solid contact ion selective electrodes (SC-ISEs) are preferred in the market rather than the liquid contact (LC) ones. They are highly compatible for mass production and can easily be miniaturized.

Although SC-ISEs function in the same fundamental way as LC-ISEs, several drawbacks can be noticed in the SC-ISEs that might limit their functionality. SC-ISEs function primarily like conventional LC-ISEs in respect of ionic sites and ionophores present in the sensing membrane. However, problems such as potential drift, can highly worsen the detection limits and hence their reliability. Recent studies mainly focus on optimization of these electrodes and reduction of their drawbacks. The reason behind these drawbacks is the formation of a water layer at the ion selective membrane (ISM)/electrode substrate interface. This water layer acts as an electrolyte reservoir where ion composition changes by even tiny transmembrane fluxes of the primary and interfering ions, which in turn affect the potential stability severely [7]. So, minimizing the water uptake is a must that could be attained upon using highly hydrophobic SC materials [8].

Studies regarding the enhancement of SC-ISEs stability focus primarily on developing the interface between the electron conducting electrode substrate and the ion conducting ISM [9]. Accordingly, the use of electrically conductive polymer (ECP) as an ion-to-electron transducer in SC-ISE emerged owing to both their electronic and ionic conductivity [10]. Polypyrrole, polyaniline and poly(3-octylthiophene) (POT) as ECP have been utilized as SC layer but these ECP-based SC-ISE suffered from deficient long-term potential stability and irreproducible standard potential. Later, carbon nanotubes [11, 12], graphene [4, 13] and other carbonaceous materials compete with ECPs due to their large surface area and capacitance, that lead to an effective stabilization of the electrode potential of SC-ISE. Being more inert than ECPs, they offer crucial advantages with respect to potential stability and avoiding the formation of the water layer at the interface. Using a composite of both ECP and graphene enhance redox capacitance as reported [14]. The investigations of water layer formation process concluded that hydrophobic surfaces are effective in exclusion of such layers this positively affects the potential stability and sensor performance. Many hydrophobic surfaces were studied, such as hydrophobic carbon nanotubes (CNTs) [15] that have been prepared by chemically modifying CNTs with octadecylamine, it was realized that those modified CNTs not only enhanced the potential stability, but furthermore, improved standard potential reproducibility (*E*^0^) [16]. Another hydrophobic surface was simply prepared by modifying Au electrode surface with perfluorodecanethiol to produce a hydrophobic self-assembled monolayer [17].

Hydrophobic conducting polymers have also been prepared, where the hydrophobicity was either inherited within the polymer structure such as polyazulene [18, 19] or induced during the polymerization process through the incorporation of hydrophobic counterions such as incorporation of perfluorooctane sulfonate [20] in polypyrrole or incorporation of tetrakis(pentafluorophenyl)borate (TFAB^–^) anion [21] in poly(3,4-ethylenedioxythiophene), or preparation of a copolymer of polyaniline with hydrophobic polystyrene by electrospinning [22]. Recently, the hydrophobic polymer poly(tetrafluoroethylene) (PTFE) has been impregnated within the ISM, which improved the potential stability [23].

**Fig. 1:**
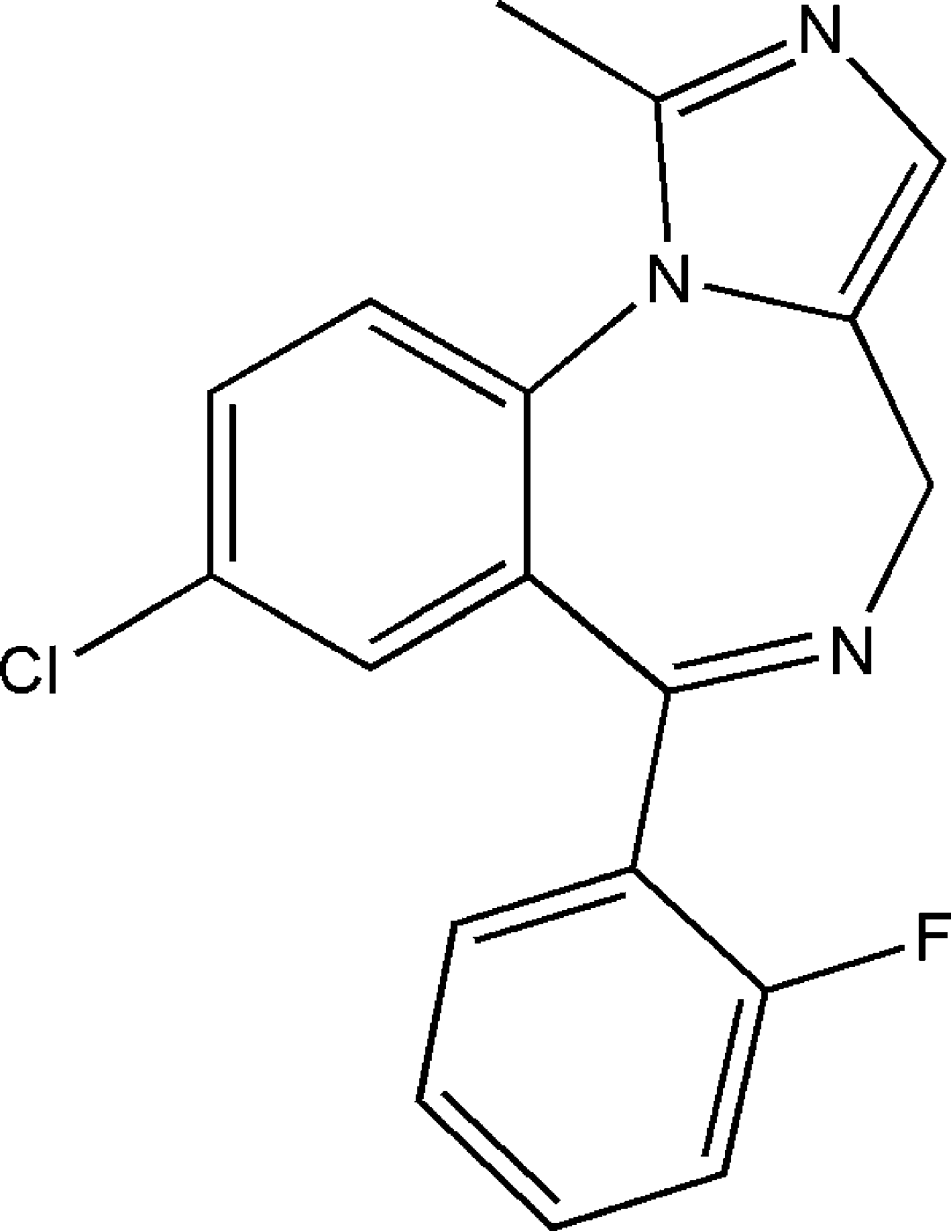
Midazolam chemical structure

In this study, glassy carbon electrode (GCE) was employed. Glassy carbon is known for its many advantages such as high heat and chemical resistance but most significant of all is its high impermeability to liquids and gases [24]. To make use of that property, GCE was used as substrate for electropolymerization of the perfluorinated aniline monomer in acidic solution using cyclic voltammetric scanning. By combining glassy carbon with in-situ polymerized ECP, we intend to eliminate water layer formation. This will result in a highly stable and reliable sensor. Having these sensors available on hand, in determination of potentially addictive drugs for example, will be highly beneficial. Several studies have been conducted recently for determination of such drugs whether in dosage form [25, 26] or biological fluids [27, 28]. Midazolam (MDZ) was chosen as a target analyte in this work, its chemical structure is presented in Fig. 1. MDZ belongs to the benzodiazepines class having hypnotic-sedative effect with anxiolytic, muscle relaxant, anticonvulsant, sedative, hypnotic, and amnesic properties.

It should be emphasized that our aim in this work is to design SC-ISEs that have improved potential stability based on perfluorinated polyaniline derivatives. The modified electrode was characterized by electrochemical impedance spectroscopy to assess the surface characteristics. It was then coated with the ionophore doped PVC ion sensing membrane and was employed for midazolam determination in its pharmaceutical formulation. The designed SC-ISE was also characterized as per IUPAC recommendations and compared to a control one. These optimized SC-ISEs can be later employed in determination of benzodiazepines whether in pharmaceutical formulations or biological fluids without prior extraction.

## Experimental

### Material and reagents

All reagents and chemicals used were of analytical grade and the water was bi-distilled. 3,5 bis(trifluoromethyl)aniline, high molecular weight polyvinyl chloride (PVC), Potassium tetrakis (4-chlorophenyl)borate (KTCPB), calix-[8]-arene (CX8), calix-[6]-arene (CX6), calix-[4]-arene (CX4), 2-hydroxypropyl β-cyclodextrin (HP-β-CD), 2-nitrophenyl octyl ether (*o*-NPOE) and midazolam (MDZ) United States Pharmacopeia (USP) Reference Standard were purchased from Sigma-Aldrich (Steinheim, Germany). Tetrapentylammonium bromide ((Pen)_4_N^+^Br^−^) was purchased from Merck (Darmstadt, Germany) while tetrahydrofuran (THF) was obtained from BDH (Poole, England). Acetate buffer pH 4 was used. Midathetic^*®*^ ampoule (5 mg/mL) was manufactured by Amoun Pharmaceutical Co. S.A.E. (Batch No. 182828). Glassy carbon electrodes were used as the electrode (3 mm diameter, CH Instruments, Texas, USA).

### Instruments

Metrohm Autolab potentiostat/galvanostat (model PGSTAT204), and a three-electrode system with Ag/AgCl double junction (Z113107-1EA), Aldrich, USA as a reference electrode and platinum counter electrode, was used. A Jenway digital ion analyzer model 3330 (Jenway, UK). A Jenway pH glass electrode (Jenway, UK), magnetic stirrer. Bandelin sonorox, Rx 510 S, magnetic stirrer (Hungarian). XPS data was collected using K-ALPHA (Thermo Fisher Scientific, USA) with monochromatic X-ray Al K-alpha radiation source, spot size 400 mm, and full spectrum pass energy 200 eV.

### Preparation of ion selective membrane (ISM)

Conventional liquid membrane ISEs were used only for screening of the most selective ionophore towards MDZ, different ionophores were added to four ISMs besides a control one (an ionophore free ISM). The ISMs cocktail (600 mg total weight) were dissolved in 6 mL THF, and the composition of membranes was summarized in the Table 1. For liquid contact sensors, the ISM was poured in Petri dish (5 cm) and left overnight to evaporate the solvent leaving a membrane of ≈ 0.1 mm thickness. From master membranes, disks (5 mm diameter) were punched and stuck using THF to a tube made of PVC. Equal volumes of 10^–4^ M MDZ and 10^–4^ M KCl were mixed preparing the inner filling solution, then Ag/AgCl wire was introduced to act as an internal reference electrode. For SC-ISEs preparation, 10 μL of the ISM of choice was drop-casted onto the PTFANI modified GCE; elecroploymerized layer on the surface of glassy carbon electrode and left overnight to evaporate.

**Table 1:** Sensors composition

	PVC (%)	NPOE (%)	K-TCPB (%)	Ionophore (%)
Control	33.17	66.6	0.23	–
CX4	32.75	66.6	0.23	0.42
CX6	32.54	66.6	0.23	0.63
CX8	31.89	66.6	0.23	1.28
Beta-CD	31.85	66.6	0.23	1.32

### Electropolymerization of 3,5 bis(trifluoromethyl)aniline

3,5 bis(trifluoromethyl)aniline monomer was electrochemically polymerized on GCE that has been previously polished with alumina, rinsed with water and dried under stream of N_2_. Polymerization was accomplished in three electrode cell assembly (Ag/AgCl reference electrode, platinum counter electrode) connected to Metrohm Autolab potentiostat/galvanostat PGSTAT204. Cyclic voltammetry in potential range 0.0 V and + 1.5 V (3 cycles, ν = 100 mV s^−1^) was used for electropolymerization 3,5 bis(trifluoromethyl)aniline in aqueous 0.1 M H_2_SO_4_ acidic solutions containing 1 mM of 3,5 bis(trifluoromethyl)aniline. H_2_SO_4_ serves two purposes here, first as an acidic medium to encourage polymerization. It also aids the miscibility of 3,5-bis(trifluoromethyl)aniline in water.

### Electrochemical characterization and measurements

For calibration curves construction, each sensor was immersed and conjugated with Ag/AgCl double junction reference electrode in MDZ solutions covering a concentration range of 1 × 10^−7^–1 × 10^−2^ M. The sensor was left to equilibrate while stirring until a constant potential reading is achieved. The potential differences were measured within ± 1 mV. Calibration plots of the electrodes were constructed relating the recorded *emf* of each sensor to the corresponding − log molar concentrations of MDZ. The selectivity of the proposed sensors was assessed by calculating the potentiometric selectivity coefficient [− log (K^Pot^_Primary ion, interferent_) to estimate the degree of interference of the foreign substance on the electrodes’ response using separate solutions method (SSM) [29].

Electrochemical impedance spectroscopy (EIS) was performed using Metrohm Autolab PGSTAT204 potentiostat/galvanostat, and a three-electrode configuration where GCE electrode, Ag/AgCl, and Pt had been used as working electrode, reference electrode, and counter electrode, respectively. The experiments were carried out in 0.1 M KCl solution at room temperature. The frequency range used was from 100,000 Hz to 100 mHz, and the applied signal was 5 mV alternating voltage. The data validity was confirmed using Kramers–Kronig (KK) transformation. To estimate the circuit components, the results were fitted to a Randles’ equivalent circuits utilizing the Nova 1.11.0 software.

### Determination of MDZ in pharmaceutical formulation

Into 25-mL volumetric flask, 1.6 mL of the Midathetic^*®*^ ampoule was accurately transferred and the volume was completed with acetate buffer pH = 4. The concentration of the prepared samples was 1 × 10^–3^ M. The potentiometric measurements were recorded using the proposed sensors and the concentration was calculated from a regression equation obtained using the calibration curves.

## Results and discussion

### ISM design, optimization, and ionophores selection

**Fig. 2:**
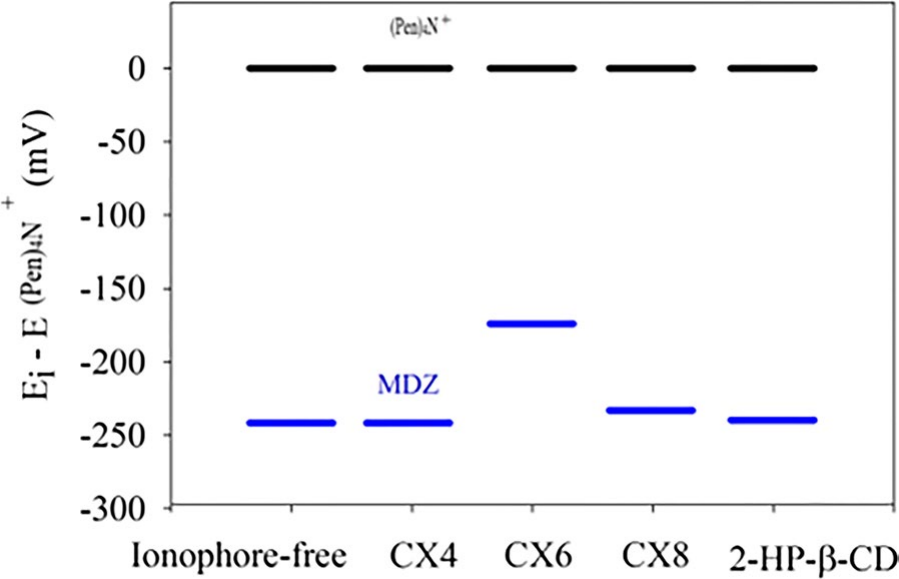
The emf response of different ISEs based on different ionophores to 1 × 10^−3^ M solutions of midazolam (after subtraction of the emf of the same ISE towards 1 × 10^−3^ M (Pen)_4_N^+^Br^−^

Efforts were exerted to improve the selectivity of the proposed ion-selective electrodes by experimenting with different ionophores. For this reason, screening of different commercially available ionophores such as calix-[8]-arene (CX8), calix-[6]-arene (CX6), calix-[4]-arene (CX4), 2-hydroxypropyl β-cyclodextrin (HP-β-CD) were investigated. Ionophores are known for providing inclusion sites which allow guest molecules to bind with, which positively impact the sensor’s selectivity. Calixarenes are basket-shaped with electron-rich cavities capable of forming inclusion complexes with various ions and molecules [30]. Cyclodextrins (CDs) act as molecular receptors which bind with guest molecules through intermolecular hydrogen bonding [31]. To ensure complexation of MDZ with the ionophore, responses of MDZ-ISEs (doped with different ionophores) to (Pen)_4_N^+^Br^−^ were recorded (Pen)_4_N^+^Br^−^ is too bulky which hinders its binding to the ionophores unlike MDZ. Besides it is highly hydrophobic which causes a strong response in the ISE. That’s why (Pen)_4_N^+^Br^−^ was selected as a reference ion, as previously employed in literature [4, 27, 32, 33]. For ensuring the most selective ionophore for MDZ the response of ISEs doped with different ionophores was compared to (Pen)_4_N^+^Br^−^. Figure 2 shows the differences in potential (*ΔE* = *E*_*MDZ *_*− E*_(Pen)4N_^+^_Br_^−^) among the *emf* values of the proposed ISEs (ionophore-free, or doped with CX4, CX6, CX8 or HP-β-CD) in response to MDZ compared to (Pen)_4_N^+^Br^−^. The minor the difference in potential compared to (Pen)_4_N^+^Br^−^ the more efficient ion transfer of MDZ into the ISM. The CX6 doped membrane showed the smallest *ΔE* (174 mV) compared to 222 mV, 241 mV, 233 mV, and 239 mV for the ionophore-free membrane, CX8, CX4 and HP-β-CD doped membranes, respectively. These results indicate that supramolecular ionophore CX6 has the maximum binding affinity towards MDZ. It should be noted that β-CD has been reported [34] as an ionophore for midazolam detection, but it did not offer better selectivity in our case. Therefore, CX6 was the ionophore of choice in conducting our studies.

### Electropolymerization of perfluorinated polyaniline on GCE

**Fig. 3:**
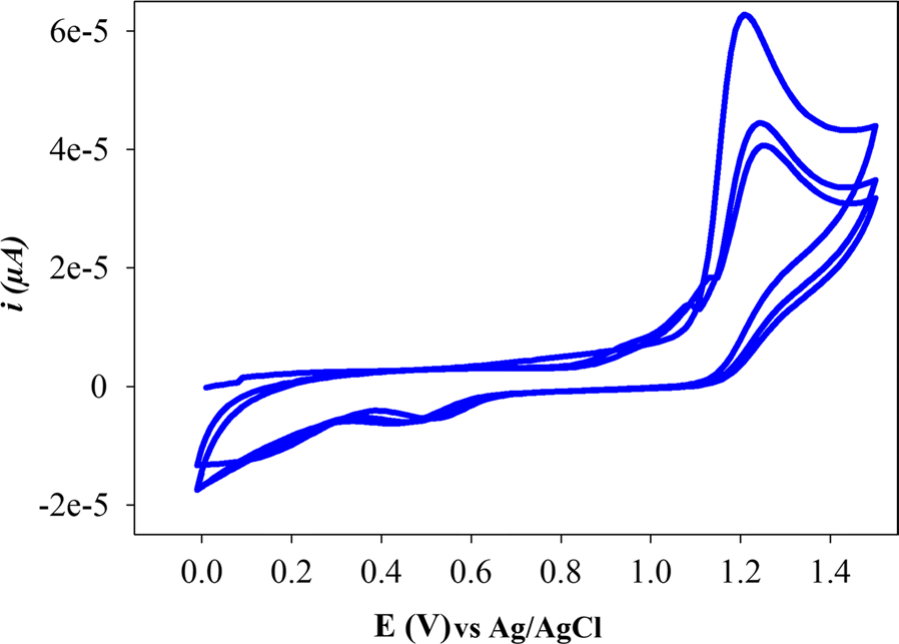
Cyclic voltamogram of three scan cycles for 1 mM 3,5 bis(trifluoromethyl)aniline in 0.1 M H_2_SO_4_ on GCE working electrode versus Ag/AgCl reference electrode

Perfluorinated polyaniline hydrophobicity is embedded within the polymer chemical structure, the fluorine has been incorporated in polyaniline structure to increase its hydrophobicity and thermal stability [35, 36]. Figure 3 shows the voltammogram during anodic polymerization of 3,5 bis(trifluoromethyl)aniline on GCE, during the first scan there is an oxidation peak about 1.2 V versus Ag/AgCl reference electrode. The oxidation peak progressively decreases after each scan and shifts to slightly positive potential. There is a reduction peak that appear at 0.2 V and grows after each cycle. This reduction peak can be attributed to the initial formation of a different reduced form followed by oxidation to another form as previously reported for polyaniline [37]. These results indicate the formation of adherent polymeric film on the surface of GCE. The potential stability of the sensors fabricated with higher cycle number was inferior to the sensors fabricated with 3 cycles. It is worth noting that hydrophobic films have been produced previously by electropolymerization of diazonium salt of 3,5-bis(trifluoromethyl)benzene diazonium salt in reductive way using multiple cycles.

### Characterization of the perfluorinated polyaniline

**Fig. 4:**
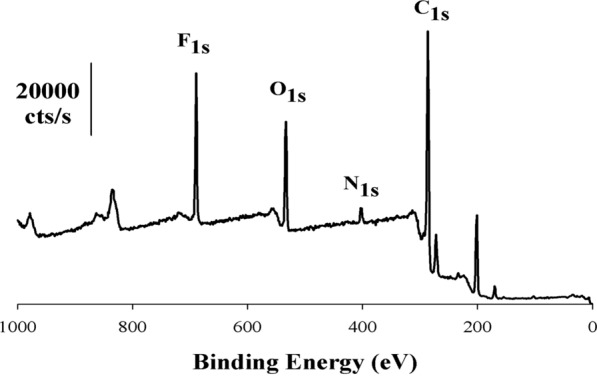
XPS survey spectrum of PTFANI on carbon electrode

To confirm that the carbon electrode surface has been modified during electropolymerization, XPS analysis was performed on the electrode after the polymerization step. Figure 4 shows a survey spectrum of carbon modified with the poly(3,5bis(trifluoromethyl)aniline (PTFANI) polymer, there are two peaks at 402.2 eV, and 689.1 eV which are attributed to presence of N(1*s*) and F(1*s*) respectively on the carbon electrode surface which has a prominent peak around 284.6 eV.

**Fig. 5:**
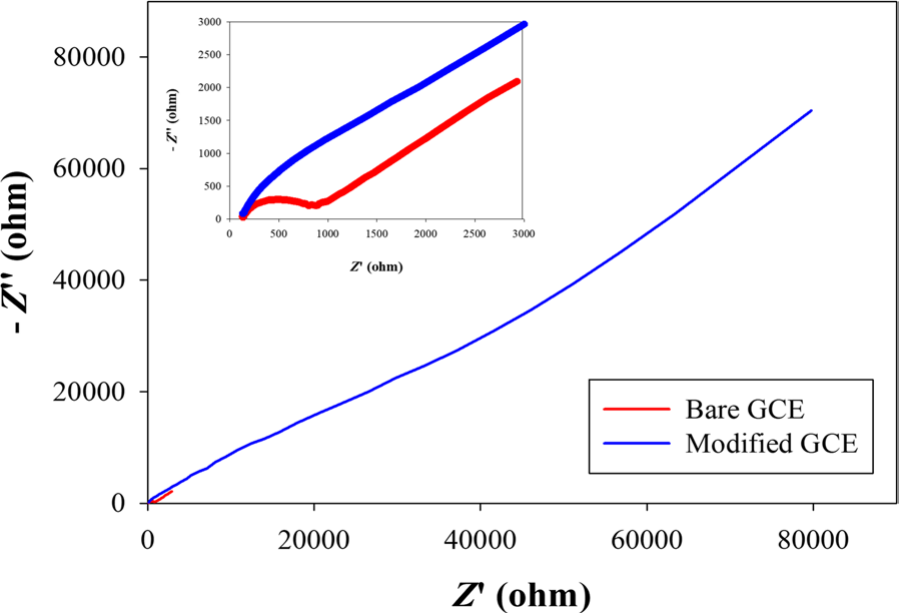
Nyquist plots of bare GCE (red curve) and perfluorinated polyaniline modified GCE (blue curve) versus Ag/AgCl reference electrode in 1 mM potassium ferrocyanide/ferrocyanide and 0.1 M KCl at frequency from 100 kHz to 0.1 Hz and 5 mV ac perturbation signal

Electrochemical Impedance spectroscopic methods has been employed to characterize the poly(3,5bis(trifluoromethyl)aniline) (PTFANI) film. EIS is a surface sensitive technique for investigating and characterizing GCE surface. The surface study of both unmodified GCE and modified GCE/PTFANI interfaces was carried out by EIS. The impedance results represented as Nyquist plots in Fig. 5. Nova software was used to fit to equivalent Randles’ circuits to estimate circuits’ parameters such solution resistance (R_s_), electrode charge transfer resistance (R_ct_), and double layer capacitance (C_dl_). For the bare GCE electrode, the figure shows a small semicircle in the Nyquist plot, and the calculated R_ct_ and C_dl_ were 597 Ω and 584 nF respectively, showing a low electron transfer resistance. On the other hand, the GCE modification with PTFANI film, resulted in increasing the semicircle diameter significantly. These results are consistent with voltammetric characterization and proves that the charge transfer resistance was increased R_ct_ (34.2 kΩ). The voltametric and EIS results provide strong evidence for the successful modification with PTFANI polymer thin layer. Moreover, this polymer film has fewer conducting properties than bare GCE. Moreover, GCE/PTFANI double layer capacitance (C_dl_) have been increased to 3.51 μF, compared to bare GCE and this capacity change can explain the observed potential stability of the sensor signal beside the hydrophobicity of the PTFANI (as shown below).

### Performance of the potentiometric sensors

**Fig. 6:**
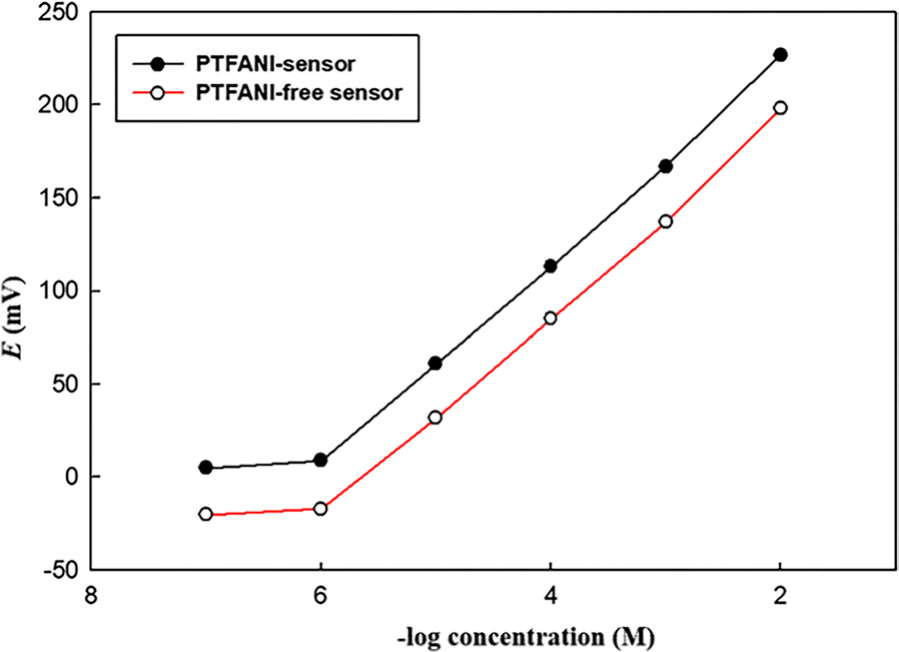
Profile of the potential in mV versus − log molar concentration of midazolam using the proposed sensors

The electrochemical performance of the proposed sensors; GCE/ISM(CX6) and GCE/PTFANI/ISM(CX6), were evaluated as per the IUPAC recommendations [38] as presented in Table 2. The SC-ISE modified PTFANI transducer layer possesses Nernstian slope (54.5 mV/decade) while PTFANI free sensor has slope equal to (53.5 mV/decade) over a concentration range of 1 × 10^–6^–1 × 10^–2^ M. Figure 6 shows the calibration plots of the proposed sensors. The effect of PTFANI layer in minimizing the potential drift was evaluated. The potential drift of GCE/ISM(CX6) sensor = 3.1 mV/h as observed for 10^–4^ M MDZ and diminished to 100 μV/h in GCE/PTFANI/ISM(CX6) sensor.

**Table 2:** Electrochemical response characteristics and validation parameters of the proposed Midazolam sensors

Parameter	PTFANI-free sensor	PTFANI-sensor
Slope (mV/decade)^a^	53.5	54.5
Intercept (mV)^a^	301.0	332.4
Correlation coefficient (r)	0.9992	0.9998
Response time (s)	25	10
Working pH range	2.5–4	2.5–4
Concentration range (M)	1 × 10^–6^–1 × 10^–2^	1 × 10^–6^–1 × 10^–2^
Stability (days)	20	30
Accuracy (mean ± SD)	100.13 ± 1.57	99.89 ± 1.13
Precision (% RSD)		
Intra-day precision^a^	0.864	1.311
Interday precision^b^	1.296	1.967
LOD (M)^c^	9.0 × 10^–7^	9.0 × 10^–7^

^a^Three different concentrations of three replicate each (n = 9) repeated three times within the same day

^b^Three different concentrations of three replicate each (n = 9) repeated on three successive days

^c^Limit of detection (measured by intersection of the extrapolated arms of non-responsive and the Nernstian segments of the calibration plot

### Effect of pH on SC-ISEs performance

**Fig. 7:**
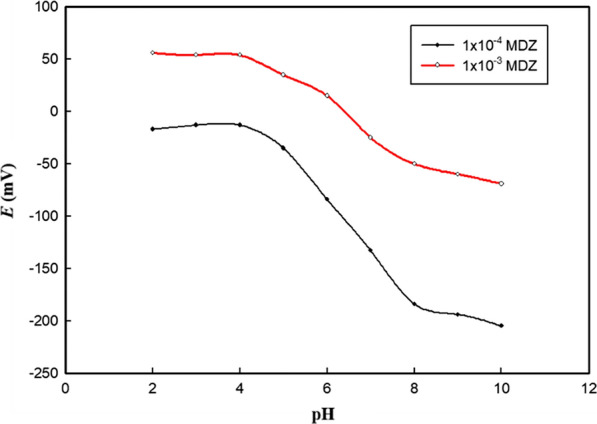
Potential pH profile for the proposed GCE/PTFANI/ISM(CX6) sensor

For experimental conditions optimization, the influence of pH on the electrode performance was investigated. Constant response was observed in pH range from 2.5 to 4 as shown in Fig. 7 while measuring the response of 1 × 10^−3^ M and 1 × 10^−4^ M MDZ.

### Potentiometric aqueous layer test

**Fig. 8:**
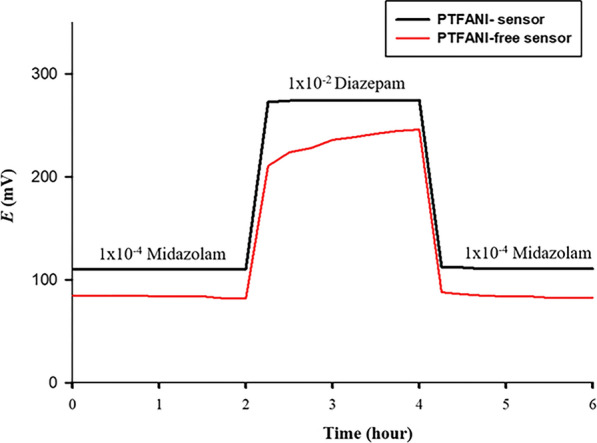
Potentiometric aqueous layer test using PTFANI-Sensor and PTFANI-free Sensor

As previously mentioned, the two inherent limitations of solid contact electrode; poor reproducibility of standard potential and potential drift are attributed to water layer formation. Long term stability of solid contact electrode was tested by applying the potentiometric aqueous layer test [39]. The test is based on measuring potential changes by altering from drug solution (1 × 10^–4^ M) to a concentrated interferent ion solution (1 × 10^–2^ M) and then measuring the drug solution again. Diazepam was chosen in this case as an interferent ion as it belongs to benzodiazepines and behaves similarly to MDZ at the selected pH. This test is based on recording potential drift when changing from MDZ (1 × 10^–4^ M) to (1 × 10^–2^ M) interfering diazepam solution and followed by MDZ again. Existence of a water layer at the interface between the solid contact and ISE membrane leads to an observable drift due to the variation of the composition of the aqueous layer due to transmembrane ion fluxes. The presence of PTFANI diminished the potential drift as observed in Fig. 8. In addition to a contact angle of 120°, this signifies the lack of the aqueous layer while an evident potential drift was observed in case of PTFANI-free sensor.

### Selectivity of the proposed sensors

Separate solutions method [29] was chosen for assessing the selectivity of the proposed sensors in the presence of expected interfering ions. Potentials were measured for 1 × 10^–3^ M MDZ solution and then for 1 × 10^–3^ M interferent solution, separately, then the following equation was used to calculate the potentiometric selectivity coefficients:

$$ - \log \,\left( {{\text{K}}_{\text{primary ion interferent}}^{{\text{pot}}} } \right) = \left( {\frac{{{\text{E}}_{\text{I}} - {\text{E}}_{{\text{drug}}} }}{{2.303 RT/Z_{drug} F}}} \right) + \left( {1 + \frac{{Z_{drug} }}{Z_I }} \right)\log \left[ {drug} \right] $$

where E_drug_ and E_I_ are the potential reading of the drug and the interferent, respectively, Z_drug_ and Z_I_ are the charges on the drug and the interfering ion, respectively and 2.303 RT/Z_drug_F represent the slope of the investigated sensor (mV/decade).

Satisfactory selectivity values showed that the proposed sensors exhibited high selectivity towards MDZ as presented in Table 3. It should be highlighted that PTFANI sensor shows better values, hence better selectivity compared to transducer free sensor.

**Table 3:** Potentiometric selectivity coefficients (− log K^pot^_midazolam, Interferent_) of the proposed sensors

Interferent (10^–3^ M)	PTFANI-free sensor	PTFANI-sensor
KCl	2.170	3.594
MgCl_2_	2.075	3.319
CaCl_2_	1.962	3.080
NaCl	1.887	2.842

### Potentiometric determination of MDZ in pharmaceutical formulations

The proposed sensors succeeded in analysis of MDZ in pharmaceutical formulation (Midathetic^®^ ampoule) as observed in Table 4. The statistical analysis of the results of pure powder indicates that there is not any difference between reported method [40] and proposed potentiometric sensor, Table 5.

**Table 4:** Determination of midazolam in Midathetic^®^ ampule using the proposed sensors

Midathetic^®^ ampule		
Parameters	PTFANI-free sensor	PTFANI-sensor
Mean^a^	99.34	100.73
± SD	1.37	0.67

^a^Average of three determination

**Table 5:** Statistical comparison of the results of the proposed sensors with the reported method

Parameters	PTFANI-free sensor	PTFANI-sensor	Reported method^a^
*n*	9	9	9
Mean	99.13	99.89	100.69
SD	1.47	1.13	0.92
Variance	2.16	1.28	0.85
*t*-test (2.12)^b^	0.968	1.64	
F (3.44)^b^	2.541	1.51	

^a^Reported HPLC method: C18 column, using a mobile phase: CAN-phosphate buffer (pH 3.3) (30:70 v/v) at a flow rate of 1.0 mL/min and UV detection at 220 nm

^b^These values represent the corresponding tabulated values of *t* and F at *p* = 0.05

## Conclusion

In this work, hydrophobic perfluorinated polyaniline has been electrochemically polymerized on the surface of GCE to fabricate a SC-ISE with enhanced potential stability. The number of polymerization cycles were found to affect the potential stability. As the number increase; the hydrophobic polymer film becomes more insulating and might negatively affect the potential stability. Three voltammetric cycles were optimal for the sensor potential stability. Electropolymerization offers precise control of polymer thickness and characteristics rather than chemical polymerization. The modified PTFANI sensor was superior in terms of potential stability (100 μV/h) versus control sensor (3.1 mV/h). Moreover, the selectivity towards midazolam was higher than that of the control sensor. The fabricated SC-ISE sensor was employed successfully to quantitate midazolam in pharmaceutical formulation without any prior steps. This modified electrode offer the possibility of benzodiazepines’ abuse detection in biological fluids. To the best of our knowledge, this polymer was not previously employed in ion selective electrodes. This greatly enhanced the stability of the designed ISE. Moreover, the designed SC-ISE is cost effective and easily portable. This coupled with its relative stability to other SC-ISEs encourages its further application in other matrices. They require no extraction unlike other conventional analytical techniques routinely utilized in benzodiazepines detection.

## Data Availability

The datasets used and/or analysed during the current study are available from the corresponding author on reasonable request.

## References

[1] Cuartero M, Bakker E (2017). Environmental water analysis with membrane electrodes. Curr Opin Electrochem.

[2] Safwat N, Mahmoud AM, Abdelghany M, Ayad MF (2021). In situ monitoring of triclosan in environmental water with subnanomolar detection limits using eco-friendly electrochemical sensors modified with cyclodextrins. Environ Sci Process Impacts.

[3] Beullens K, Mészáros P, Vermeir S, Kirsanov D, Legin A, Buysens S, Cap N, Nicolaï BM, Lammertyn J (2008). Analysis of tomato taste using two types of electronic tongues. Sens Actuators B Chem.

[4] El-Mosallamy SS, Ahmed K, Daabees HG, Talaat W (2020). A microfabricated potentiometric sensor for metoclopramide determination utilizing a graphene nanocomposite transducer layer. Anal Bioanal Chem.

[5] Merey HA, El-Mosallamy SS, Hassan NY, El-Zeany BA (2020). Green monitoring of bromhexine oxidative degradation kinetics. Microchem J.

[6] El-Sayed G, El Mously D, Mostafa N, Hassan N, Mahmoud A (2021). Design of copper microfabricated potentiometric sensor for in-line monitoring of neostigmine degradation kinetics. Electroanalysis.

[7] Fibbioli M, Morf WE, Badertscher M, de Rooij NF, Pretsch E. Potential drifts of solid‐contacted ion‐selective electrodes due to zero‐current ion fluxes through the sensor membrane. Electroanalysis Int J Devoted Fundam Pract Aspects Electroanal. 2000;12:1286–92.

[8] Sundfors F, Lindfors T, Hofler L, Bereczki R (2009). Gyurcsányi RbE: FTIR-ATR study of water uptake and diffusion through ion-selective membranes based on poly (acrylates) and silicone rubber. Anal Chem.

[9] Paciorek R, van der Wal PD, de Rooij NF, Maj‐Żurawska M. Optimization of the composition of interfaces in miniature planar chloride electrodes. Electroanal Int J Devoted Fundam Pract Aspects Electroanal. 2003;15:1314–8.

[10] Bobacka J. Conducting polymer‐based solid‐state ion‐selective electrodes. Electroanal Int J Devoted Fundam Pract Aspects Electroanal. 2006;18:7–18.

[11] Mahmoud AM, Saad MN, Elzanfaly ES, Amer SM, Essam HM (2020). An electrochemical sensing platform to determine tetrahydrozoline HCl in pure form, pharmaceutical formulation, and rabbit aqueous humor. Anal Methods.

[12] Hassan SA, Nashat NW, Elghobashy MR, Abbas SS, Moustafa AA, Mahmoud AM (2022). Novel microfabricated solid-contact potentiometric sensors doped with multiwall carbon-nanotubes for simultaneous determination of bisoprolol and perindopril in spiked human plasma. Microchem J.

[13] Mahmoud AM, Ragab MT, Ramadan NK, El-Ragehy NA, El-Zeany BA (2020). Design of solid-contact ion-selective electrode with graphene transducer layer for the determination of flavoxate hydrochloride in dosage form and in spiked human plasma. Electroanalysis.

[14] Chen W, Rakhi R, Alshareef HN (2013). Capacitance enhancement of polyaniline coated curved-graphene supercapacitors in a redox-active electrolyte. Nanoscale.

[15] Yuan D, Anthis AH, Ghahraman Afshar M, Pankratova N, Cuartero M, Crespo GNA, Bakker E. All-solid-state potentiometric sensors with a multiwalled carbon nanotube inner transducing layer for anion detection in environmental samples. Anal Chem. 2015;87:8640–5. 10.1021/acs.analchem.5b01941 26272001

[16] Papp S, Kozma J, Lindfors T, Gyurcsányi RE (2020). Lipophilic multi-walled carbon nanotube-based solid contact potassium ion-selective electrodes with reproducible standard potentials. A comparative study. Electroanalysis.

[17] Abd El-Rahman MK, Abou Al-Alamein AM, Abdel-Moety EM, Fawaz EM (2017). Integrated gold-thiol based potentiometric sensors for in situ dual drug-protein binding studies on naproxen/diphenhydramine salts model. J Electrochem Soc.

[18] He N, Höfler L, Latonen R-M, Lindfors T (2015). Influence of hydrophobization of the polyazulene ion-to-electron transducer on the potential stability of calcium-selective solid-contact electrodes. Sensors Actuators B Chem.

[19] He N, Gyurcsányi RE, Lindfors T (2016). Electropolymerized hydrophobic polyazulene as solid-contacts in potassium-selective electrodes. Analyst.

[20] He N, Papp S, Lindfors T, Höfler L, Latonen R-M (2017). Gyurcsányi RbE: pre-polarized hydrophobic conducting polymer solid-contact ion-selective electrodes with improved potential reproducibility. Anal Chem.

[21] Papp S, Bojtár M, Gyurcsányi RBE, Lindfors T. Potential reproducibility of potassium-selective electrodes having perfluorinated alkanoate side chain functionalized poly(3,4-ethylenedioxytiophene) as a hydrophobic solid contact. Anal Chem. 2019;91:9111–8. 10.1021/acs.analchem.9b01587 PMC6750645 31184105

[22] Jiang W, Liu C, Zhao Y, Waterhouse GI, Zhang Z, Yu L (2019). A solid-contact Pb^2+^-selective electrode based on a hydrophobic polyaniline microfiber film as the ion-to-electron transducer. Synth Met.

[23] Fan Y, Huang Y, Linthicum W, Liu F, Beringhs AOR, Dang Y, Xu Z, Chang S-Y, Ling J, Huey BD (2020). Toward long-term accurate and continuous monitoring of nitrate in wastewater using poly (tetrafluoroethylene) (PTFE)—solid-state ion-selective electrodes (S-ISEs). ACS Sens.

[24] Desimoni E, Brunetti B (2012). Glassy carbon electrodes film-modified with acidic functionalities. A review. Electroanalysis.

[25] Dahham QF, Ahmed ED (2020). Spectrophotometric determination of Diazepam and Propranolol hydrochloride in pharmaceutical by dual wavelengths method. Samarra J Pure Appl Sci.

[26] Vahidifar M, Es’haghi Z. Magnetic nanoparticle-reinforced dual-template molecularly imprinted polymer for the simultaneous determination of oxazepam and diazepam using an electrochemical approach. J Anal Chem. 2022;77:625–39.

[27] Hassan SA, ElDin NB, Zaazaa HE, Moustafa AA, Mahmoud AM (2020). Point-of-care diagnostics for drugs of abuse in biological fluids: application of a microfabricated disposable copper potentiometric sensor. Microchim Acta.

[28] Nakhodchi S, Alizadeh N (2021). Rapid simultaneous determination of ketamine and midazolam in biological samples using ion mobility spectrometry combined by headspace solid-phase microextraction. J Chromatogr A.

[29] Ma TS, Hassan SS. *Organic analysis using ion-selective electrodes.* London: Academic Press; 1982.

[30] Namor MLDD, Cleverley AF, Zapata-Ormachea RM. Thermodynamics of calixarene chemistry. Chem Rev. 1998; 98:2495–526. 10.1021/cr970095w 11848969

[31] Carneiro SB, Fernanda Í, Duarte C, Heimfarth L. Cyclodextrin-drug inclusion complexes: in vivo and in vitro approaches. Int J Mol Sci. 2019;20:1–23. 10.3390/ijms20030642 PMC6387394 30717337

[32] Chen Q, Yang L-P, Li D-H, Zhai J, Jiang W, Xie X (2021). Potentiometric determination of the neurotransmitter acetylcholine with ion-selective electrodes containing oxatub [4] arenes as the ionophore. Sens Actuators B Chem.

[33] Mousavi MP, Abd El-Rahman MK, Mahmoud AM, Abdelsalam RM, Bühlmann P (2018). In situ sensing of the neurotransmitter acetylcholine in a dynamic range of 1 nM to 1 mM. ACS Sens.

[34] Amorim C, Araújo A, Montenegro M, Silva V (2008). Cyclodextrin-based potentiometric sensors for midazolam and diazepam. J Pharmaceut Biomed Anal.

[35] Waware U, Summers GJ, Hamouda A, Rashid M (2018). Synthesis and characterization of polyaniline, poly(3-fluoroaniline), and poly(aniline-co-3-fluoroaniline) derivatives obtained by chemical oxidative polymerization methods. Polym Plast Technol Eng.

[36] Tomšík E, Dallas P, Šeděnková I, Svoboda J, Hrubý M (2021). Electrochemical deposition of highly hydrophobic perfluorinated polyaniline film for biosensor applications. RSC Adv.

[37] Saouti F, Belaaouad S, Cherqaoui A, Naimi Y (2021). Polyaniline thin film prepared by electrochemical polymerization method. Biointerface Res Appl Chem.

[38] IUPAC. Analytical chemistry division, comission on analytical nomenclature. Pure Appl Chem. 2000;72:1851–2082.

[39] Marco RD, Veder JP, Clarke G, Nelson A, Prince K, Pretschc E, Eric B (2008). Evidence of a water layer in solid-contact polymeric ion sensors. Phys Chem Chem Phys.

[40] de Diego M, Godoy G, Mennickent S (2007). Chemical stability of midazolam injection by high performance liquid chromatography. J Sep Sci.

